# Responses to increased dietary contents of non-structural carbohydrates in herbage-based diets for dairy cows: Between- and within-cow ruminal pH variation

**DOI:** 10.1016/j.vas.2026.100614

**Published:** 2026-03-06

**Authors:** Anna-Maria Reiche, Andreas Münger, Frigga Dohme-Meier

**Affiliations:** Ruminant Nutrition and Emissions, Agroscope, Route de la Tioleyre 4, 1725 Posieux, Switzerland

**Keywords:** Non-structural carbohydrates, Subacute ruminal acidosis, SARA susceptibility, Ruminal ph, Rumen-cannulated cow

## Abstract

While dietary factors, especially high starch intake, are known to promote subacute ruminal acidosis (SARA), individual cows may differ markedly in their susceptibility and response – yet these inter- and intra-animal variations remain poorly understood, limiting targeted management. We investigated the effects of herbage-based diets with moderate (M-TNC) and high (H-TNC) levels of total non-structural carbohydrates (TNC) on ruminal fluid composition, pH, and performance of dairy cows, and their variation between and within animals. Three consecutive crossover experiments were conducted involving 14 animals. In experiments 1 and 3, cows were fed hay and concentrates (experiment 1: 23 (M-TNC) and 35 (H-TNC) % TNC; experiment 3: 18% and 36%). In Experiment 2, cows received either M-TNC (12% TNC) or H-TNC (23% TNC) fresh herbage without concentrate. The overall effects of the H-TNC diet were a greater TNC intake, a shift toward a propionate-enhanced ruminal fermentation profile, and a lower minimum ruminal pH compared to the M-TNC diet. A great between-animal variation in area under the curve at pH <5.8 (AUC_pH<5.8_) was observed: two-thirds of cows showed increased AUC_pH<5.8_ with the H-TNC diet, and one-third showed decreased or similar values. Half of the cows included repeatedly in at least two experiments consistently had a greater AUC_pH<5.8_ with the H-TNC diet than with the M-TNC diet, while the other half showed alternating reactions. Ruminal pH reactions to great dietary TNC contents seemed to vary with diet, individual cow, and time; however, larger studies are needed to confirm these interactions.

## Highlights

• Between- and within-cow variation in responses to high TNC diets were studied.
• Ruminal pH depended on TNC intake, but also on individual cow variation.
• Two-thirds of cows showed ruminal acidification with higher TNC intake.
• One-third of cows maintained or increased ruminal pH despite higher TNC intake.
• Half the cows showed varying ruminal pH responses across repeated trials.

## Graphical abstract

**Figure:**
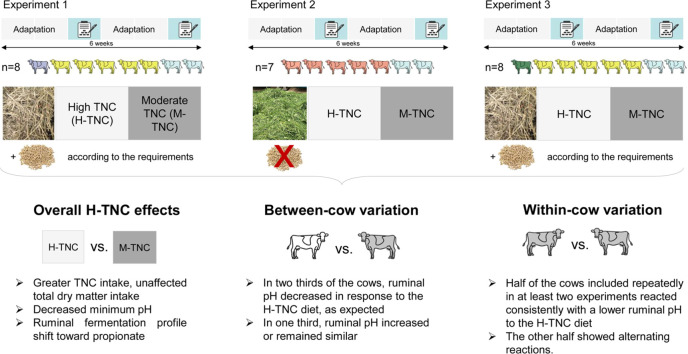

## 1 Introduction

The digestive system of ruminants is adapted to herbage-based diets. Ruminants are able to digest structural carbohydrates, such as cellulose and hemicellulose, resources that are human-inedible. Compared to other forages and feed, herbage-based diets often lack energy for high-yielding dairy cows (Kolver & Muller, 1998), requiring concentrate supplementation. By choosing selected cultivars, particular growth conditions, or both, herbage can also accumulate great contents of water-soluble carbohydrates (WSC), that is, glucose, fructose, sucrose, and fructans (Eagles, 1967; Kagan et al., 2018), allowing such herbage to boost energy intake and reduce reliance on concentrates. Along with starch, WSC contribute to total non-structural carbohydrates (TNC). Rapidly fermentable TNC may increase the risk of subacute rumen acidosis (SARA) (Abdela, 2016; Klevenhusen & Zebeli, 2021). SARA is one of the most prevalent metabolic diseases in dairy cows and occurs when ruminal pH drops below 5.5–5.8 for several hours daily (Humer et al., 2018). It can cause reduced feed intake, diarrhoea, inflammation, lameness (Plaizier et al., 2008), and reproductive disorders, leading to major economic losses (reviewed by Abdela (2016)). Concerning the influence of the individual TNC fractions on SARA, it is widely accepted that an increasing intake of starch increases the risk, whereas increasing WSC intake may increase, decrease, or not alter the ruminal pH, depending on the WSC composition (such as the degree of polymerisation) (reviewed by Klevenhusen & Zebeli, 2021). In addition to dietary factors, animal-related factors may also play a role in the manifestation of SARA. In dairy cows, a greater susceptibility to SARA has been associated with a lower lactation number and therefore lower body weight and rumen size (Neubauer et al., 2020), and with increased dry matter intake (DMI) (reviewed by Krause & Oetzel, 2006). However, previous experience with SARA can modify individual SARA susceptibility, as shown by repeated acidosis challenges in dairy cows (Dohme et al., 2008). In general, in contrast to the well-documented dietary factors – especially starch – on SARA development, the variation in ruminal pH responses between animals, and especially within the same animal over time, remains poorly studied. Therefore, this study aimed to investigate these inter- and intra-animal variations in comparison to dietary effects in dairy cows fed herbage-based diets. Specifically, we investigated (i) in a conventional manner, how increasing the TNC contents of hay- and freshly cut herbage-based diets affects the ruminal fermentation and animal performance, and (ii) as a novel aspect, the inter- and intra-individual differences in cows’ reactions to increased TNC contents involved in repeated experiments. We hypothesised that ruminal pH reactions would differ between cows, but would show intra-individual consistency over time.

## 2 Material and methods

The present study includes data from three experiments (i.e., experiments 1, 2, and 3), each conducted in a separate year over a three-year period, with lactating rumen-cannulated dairy cows at Agroscope Posieux, Switzerland. The experiments were authorised by the Animal Care Committee of the Canton Fribourg, Fribourg, Switzerland. (National No 18,630, 19,471, and 21,789). In each experiment, two forage-based diets differing in TNC content were fed to the cows.

### 2.1 Animals and housing

The experiments were conducted with 14 Holstein/Red Holstein dairy cows in total, with seven animals participating in two experiments (Exp 1 and Exp 2), and two animals participating in all three experiments (Exps 1, 2, and 3). Thus, Exp 1 involved eight animals, Exp 2 involved seven animals, and Exp 3 involved eight animals. All cows were in at least their second lactation and fitted with a rumen cannula (Bar Diamond Inc., Parma, ID, USA). Animal characteristics are shown in Table 1. The animals were kept in a tie-stall barn with stalls floored with rubber mats and sawdust and chopped straw bedding. The feed bunk space was individually separated. The animals were moved from the tie-stall barn into a milking parlour for milking (twice daily at 0500 h and 1600 h) and to an exercise yard for 1 h per day.

**Table 1:** Average production characteristics of cows at the start of the three experiments (Exp).

	Lactation Number	Days in milk	Body weight	Milk yield	Milk fat	Milk protein	Milk lactose
			(kg)	(kg/d)	( %)	( %)	( %)
Exp 1 (n = 8)	4.3 ± 2.3	54 ± 13.6	635 ± 90	34.9 ± 9.8	4.5 ± 0.44	3.2 ± 0.24	4.7 ± 0.13
Exp 2 (n = 7)	4.4 ± 2.3	258 ± 13.5	669 ± 63	21.2 ± 4.4	4.5 ± 0.83	3.6 ± 0.22	4.5 ± 0.34
Exp 3 (n = 8)	3.4 ± 1.6	74 ± 17.8	628 ± 51	37.8 ± 4.3	4.3 ± 0.38	2.9 ± 0.18	4.7 ± 0.14

### 2.2 Study design

The three experiments were set up as 2 × 2 crossover designs and lasted six weeks each. Each experiment was divided into two experimental periods, with each period including a two-week adaptation to the diet, followed by one week of data and sample collection (sampling week). In each experiment, cows were paired according to daily milk yield, days in milk (DIM), and body weight, with mean within-pair differences of 4.35 kg, 18.1 d, and 41 kg, respectively, and with the daily milk yield having the largest weight for pairing. Out of every pair, one cow was randomly assigned to one of two diets: a moderate-sugar (M-TNC) or a high-sugar (H-TNC) diet. The M-TNC diet was based on forage with ethanol-soluble carbohydrate (ESC) content aimed at the recommended level of ≤ 75 mg/kg dry matter (DM; DLG-Information, 2001). The H-TNC diet was based on forage with ESC content widely exceeding the recommended ESC level.

In Exps 1 and 3, the used forage type was hay, whereas in Exp 2, freshly cut and barn-fed herbage was used. The hay in Exps 1 and 3 originated from mixed leys with a dominance of grasses (>70 %), mainly perennial ryegrass (>50 %), red and white clovers, and forbs. High-TNC hay was from the first cut of the growth season, and M-TNC hay was from the third or fourth cuts. In Exp 2, the freshly cut herbage was harvested from an overwintered stand of Italian ryegrass (H-TNC) and from a ley with a dominance of grasses, mainly orchard grass (M-TNC). All experimental cows received additional energy and protein concentrate and mineral feed (Exps 1 and 3) or mineral feed only (Exp 2) according to their estimated requirements (Agroscope, 2021). In Exp 1, the forage-to-concentrate ratio was 84:16, whereas in Exp 3 the ratios were 78:22 (H-TNC) and 76:24 (M-TNC). Energy concentrate was composed of meals of barley (33%), wheat (31%), maize grain (31%), molasses (3%), and minerals (2%), and protein concentrate contained soybean and rape expellers (60%), maize gluten feed (25%), potato protein (10%) and molasses (5%). The mineral feed contained dicalcium phosphate, whole corn plant, livestock salt, wheat bran, calcium carbonate, mixed fat, magnesium oxide, and vitamin and trace element premix.

The results of the chemical analyses and estimated nutritive values of forages and concentrates are shown in Table 2.

**Table 2:** Chemical composition and calculated energy and protein supply of the diet ingredients.

	Experiment 1					Experiment 2			Experiment 3			
	Hay		Energy supplement n = 2	Protein supplement n = 2		Freshly cut herbage			Hay		Energy supplement n = 2	Protein supplement n = 2
	H-TNC n = 6	M-TNC n = 6				H-TNC n = 28	M-TNC n = 28		H-TNC n = 4	M-TNC n = 4		
Item (in g/kg DM unless otherwise specified)												
Dry matter (g/kg)	873	901	869	889		178	180		901	888	868	882
Organic matter	923	901	955	948		814	754		935	894	955	945
Crude protein	144	147	106	534		153	146		128	176	118	531
ADF	222	291	41	119		205	225		226	291	40	95
NDF	410	473	129	197		338	350		423	507	143	166
ESC	159	102	37	102		129	75		171	88	38	99
WSC	309	163	62	116		236	140		318	116	65	119
Starch	-	-	628	104		-	-		-	-	570	67
TNC	309	163	690	220		236	140		318	116	635	186
Calculated energy and protein content †												
NEL (MJ/kg DM)	6.3	5.2	8.1	8.5		6.1	5.2		5.7	5.7	8.1	8.5
APD ( % of DM)	99	89	102	333		99	87		90	98	101	339

Abbreviations: DM: dry matter, ADF: acid detergent fibre, NDF: neutral detergent fibre, ESC: ethanol-soluble carbohydrates, WSC: water-soluble carbohydrates, TNC: total non-structural carbohydrates, NEL: net energy of lactation, APD: Absorbable protein at the duodenum.

†Calculated according to Agroscope (2021).

In the first adaptation week, forage was offered ad libitum. During the second adaptation week and the sampling week, the amount of forage offered depended on the cow pair; that is, it equalled the mean forage intake of the pair’s cow that had ingested less during the first adaptation week plus 10 % orts. The individual daily amounts of forage and concentrate were split into two equal amounts, offered after each milking, that is, at 0800 h in the morning and at 1700 h in the afternoon. The concentrates were offered first before offering the forage. Throughout the day and evening, the forage was repeatedly pushed back into the cows’ reach. Samples of feed components were taken daily and pooled per week for analysis.

### 2.3 Measurements

Feed intake was determined daily by weighing the amount of offered feed and orts (platform scale ID-7, Mettler-Toledo, Greifensee, CH). Individual milk yields were automatically registered daily (pulsameter 2, SAC, Kolding DK). During the sampling weeks, milk samples (roughly 150 ml) were collected daily during the morning and afternoon milking for analysis of milk components. Milk samples from the morning and the afternoon of the same experimental day were stored at 4 °C until the following day, on which the two samples were manually shaken and then pooled to a total of 100 mL in aliquot relative to the respective morning and evening milk yields. Out of the 100 mL, 55 mL were conserved using a Broad Spectrum Microtab (Gerber Instruments AG, Zurich, Switzerland) and analysed for milk composition. Body weight was recorded automatically after each milking using a gated walkover scale (RIC, Insentec, Hokofarm Group, Marknesse, Netherlands). Individual ruminal pH was measured every sampling week for three consecutive days (approximately 70 h) using the Lethbridge Research Center ruminal pH measurement system (LRCpH; DASCOR, Escondido, USA). The pH measurement devices were used as described in Falk et al. (2016). They were calibrated before and after each 3-day measuring period, weighed down and connected to the plug of the cannula, and deployed into the rumen between 0630 and 0700 h on the first day of the 3-day sampling period. The pH was measured every 30 s. Ruminal fluid was collected on one afternoon of the sampling week through a rumen cannula from the ventral rumen using a 120 ml syringe and tube equipped with a terminal tubular 1-mm pore size strainer tip. Approximately 150 mL to 200 mL of ruminal fluid were collected into a flask and immediately placed on crushed ice until further processing. The ruminal fluid was homogenised by slightly inverting the collection tube and afterwards acidified as follows: For volatile fatty acid analysis, 20 mL of ruminal fluid were mixed with 0.4 mL of 50 % (m/v) sulfuric acid and split into two aliquots of 10 mL; for ammonia analysis, another 2 × 10 mL of ruminal fluid were mixed each with 0.2 mL of 50 % (m/v) trichloroacetic acid. Samples were frozen at –20 °C until analysis.

### 2.4 Laboratory analyses

The DM and organic matter contents of feed samples were analysed gravimetrically after oven drying at 105 °C for 3 h and 550 °C for 4 h. Feed crude protein (CP) content was calculated by multiplying the analysed total N content (Kjeldahl method, AOAC International, 1995, procedure 988.05) with 6.25. The acid detergent fibre (ADF) and neutral detergent fibre (NDF) contents were determined using an Ankom fibre analyser (Ankom Technology Corporation, Fairport, NT, ISO 16,472:2006 for NDF and ISO 13,906:2008 for ADF). The ethanol- and water-soluble carbohydrate contents were analysed following Hall et al. (1999) and starch content by a polarimetric method (ISO 6493 Ed. 2000–02–01). The net energy of lactation (NEL) and metabolizable protein were calculated following a previous study (Agroscope, 2021). Fat, protein, lactose, and urea contents and the somatic cell counts of the milk were analysed by Fourier-transformed IR spectrometry (Milkoscan FT 6000, Foss, Hillerød, Denmark). The analysis of volatile fatty acids and ammonia of ruminal fluid was carried out as described by Grosse Brinkhaus et al. (2016).

### 2.5 Statistical analysis of data

The intake of TNC was calculated by summing the WSC and starch intake. Any drift from the starting to the final calibration of the pH probe was assumed to be linear, and recorded data were corrected for this drift and transformed into pH units (Penner et al., 2006). A 24-h pH measurement dataset was defined as a unit. Every unit was visually plotted and subjectively validated for erroneous measurements. To calculate the duration in hours per day at which the ruminal pH was below a specified threshold, all recorded pH data points of one unit below that threshold were multiplied by the time interval of one measurement (30 s), added, and converted into hours. The pH area under the curve (AUC) at which the ruminal pH was below a threshold (<5.8) was calculated as the pH data points below the threshold times 30 s. Statistical analysis was performed using the statistic software R v.4.1.2, particularly the package NLME (linear and nonlinear mixed effects models). For analysis, measurements related to feed intake, milk production, and ruminal pH were averaged by sampling week and cow. Analyses were performed separately for each experiment and considered the 2 × 2 crossover design using a linear mixed model. Diet (H-TNC vs. M-TNC) was set as fixed effect, the animal, experimental period (1 vs. 2), and crossover sequence (1 [H-TNC→M-TNC] vs. 2 [M-TNC→H-TNC]), as random effects. Pearson correlations were computed to investigate relationships between measured variables, both using the whole dataset and subsets of M-TNC and H-TNC animals. To study between- and within-animal variations in ruminal pH reactions to the diets, data were visualised by boxplots, bar plots, and principal component analyses (PCA) using the original data, as well as by the measured variables using the individual changes between diets (ΔH-TNC - M-TNC). Finally, based on the assumption that an increased AUC_pH<5.8_ of the ruminal pH may indicate a SARA-related pH decrease (McCann et al., 2016), the reactions of animals with increased AUC_pH<5.8_ in response to the H-TNC diet were classified as ‘expected’; otherwise, they were classified as ‘unexpected’. Differences between these two subgroups of animals were investigated descriptively by graphical visualisation and were presented when visual inspection indicated marked differences.

## 3 Results

### 3.1 Comparison between moderate- and high-sugar diets

#### 3.1.1 Diet composition (descriptive comparison)

Across experiments, H-TNC diets had similar forage-to-concentrate ratios, DM, and contents of CP – except for Exp 3 where CP was lower in H-TNC hay – and APD compared to M-TNC diets (Table 2). The H-TNC diets had lower ADF and NDF and higher ESC, WSC, TNC, and NEL – except for Exp 3 where NEL contents were similar for H-TNC and M-TNC diets – contents than the M-TNC diets (Table 2).

#### 3.1.2 Feed intake, body weight, and milk production

Across all experiments, the experimental diets did not influence the total and forage intake (*p* ≥ 0.37; Table 3). Cows receiving the H-TNC diet had a greater TNC (*p* < 0.001) and lower NDF intake (all *p* ≤ 0.004; except Exp 2, *p* = 0.35) than those receiving the M-TNC diet. In Exp 3, the CP intake was greater for M-TNC cows than for H-TNC cows (*p* < 0.001), while it was not influenced by diet in Exp 1 and Exp 2 (*p* ≥ 0.19). In Exp 1, the body weight of the H-TNC cows was slightly lower (*p* < 0.010) than that of the M-TNC cows, while it was not influenced by the experimental diet in the other experiments. Daily milk yield (*p* ≤ 0.015) and milk protein percentage (*p* ≤ 0.006) were and tended to be greater for H-TNC cows than for M-TNC cows in the hay-based experiments (Exp 1 and Exp 3), and were not altered by diet in the freshly cut herbage-based experiment (Exp 2). The milk fat percentage tended to be greater for M-TNC cows than for H-TNC cows in Exp 1 (*p* ≤ 0.093) and Exp 2 (*p* = 0.038).

**Table 3:** Effects of the experimental diet on intake, body weight, milk production and ruminal fluid composition by experiment.

	Experiment 1 (n = 8)						Experiment 2 (n = 7)						Experiment 3 (n = 8)				
	Diet		SEM	P value			Diet		SEM	P value			Diet		SEM	P value	
	H-TNC	M-TNC		Diet			H-TNC	M-TNC		Diet			H-TNC	M-TNC		Diet	
																	
Body weight	663	670	44.9	0.010			676	677	45.6	0.63			654	659	33.0	0.38	
Feed intake (in kg/d unless otherwise specified)																	
Total dry matter intake	20.9	20.9	1.23	0.90			18.7	19.3	1.36	0.37			21.4	21.2	0.73	0.80	
Forage intake	17.6	17.5	0.65	0.73			18.4	19.1	1.36	0.37			16.5	16.2	0.73	0.63	
Concentrate intake^†^	3.32	3.48	0.51	0.16			0.27	0.27	0.00	^‡^			4.60	5.00	0.05	-	
Energy supplement	2.56	2.68	0.44	0.16			-	-	-	-			3.70	4.90	0.15	-	
Protein supplement	0.49	0.52	0.14	0.18			-	-	-	-			0.80	-	0.00	^‡^	
Minerals	0.27	0.28	0.01	0.16			0.27	0.27	0.00	^‡^			0.10	0.10	0.00	^‡^	
NDF intake	7.67	8.74	0.28	<0.001			6.09	6.32	0.45	0.24			9.05	10.3	0.40	0.004	
WSC intake	5.65	3.08	0.35	<0.001			4.31	2.41	0.30	<0.001			6.23	2.37	0.51	<0.001	
Starch intake	1.67	1.75	0.29	0.16			-	-	-	-			1.34	1.40	0.09	<0.001	
TNC intake	7.32	4.83	0.72	<0.001			4.31	2.41	0.24	<0.001			7.57	3.78	0.21	<0.001	
Crude protein intake	3.08	3.14	0.42	0.19			2.75	2.80	0.39	0.94			3.49	3.79	0.27	<0.001	
Milk production																	
Milk yield	32.2	30.1	4.67	0.015			16.5	15.7	3.17	0.51			33.9	31.1	1.50	0.011	
Milk fat ( %)	3.92	4.12	0.28	0.075			4.25	4.6	0.53	0.046			3.93	4.04	0.21	0.19	
Milk protein ( %)	3.32	3.21	0.10	0.002			3.59	3.53	0.21	0.10			3.27	3.09	0.06	0.005	
Milk lactose ( %)	4.75	4.71	0.08	0.12			4.25	4.25	0.19	0.44			4.68	4.66	0.06	0.49	
Ruminal fluid composition (in mol % unless otherwise specified)																	
Total volatile fatty acids (mmol/L)	138	129	5.06	0.044			126	121	4.73	0.15			114	126	5.09	0.022	
Acetate (mol %)	57.5	68.1	0.90	<0.001			63.2	67.2	0.76	<0.001			62.6	68.5	0.75	<0.001	
Propionate (mol %)	21.4	17.8	0.73	0.002			19.5	16.9	0.61	0.003			19.1	16.9	0.68	0.001	
Iso-butyrate (mol %)	0.40	0.50	0.02	<0.001			0.71	0.94	0.06	0.018			0.59	0.89	0.02	<0.001	
Butyrate (mol %)	16.6	11.9	0.92	0.002			14.0	12.2	0.30	0.001			14.6	11.1	0.41	<0.001	
Iso-valerate (mol %)	0.50	0.50	0.04	0.85			0.88	1.20	0.10	0.055			0.75	1.17	0.05	<0.001	
Valerate (mol %)	2.06	1.02	0.11	<0.001			1.16	1.05	0.06	0.030			1.78	1.00	0.18	0.002	
Acetate:Propionate	2.70	3.85	0.14	<0.001			3.25	4.03	0.17	0.003			3.34	4.09	0.19	0.001	
Ammonia (mmol/L)	2.58	3.35	0.61	0.20			5.91	11.1	1.32	0.002			4.56	7.52	0.60	0.003	
Bicarbonate (mmol/L)	0.01	0.01	0.00	0.23			0.02	0.02	0.00	0.18			0.06	0.04	0.01	0.013	

Abbreviations: H-TNC: high sugar, M-TNC: medium sugar, SEM: standard error of the mean, DM: dry matter, ADF: acid detergent fibre, NDF: neutral detergent fibre, TNC: total non-structural carbohydrates, WSC: water soluble carbohydrates, *Significant effect of period, **Significant effect of sequence. Experiment 1 and 3: hay-based diet with concentrate supplementation; experiment 2: freshly cut herbage-based diet without concentrate supplementation.

†including minerals.

‡ Model does not converge.

#### 3.1.3 Ruminal fluid composition

Across all experiments, the ruminal fluid of cows receiving the H-TNC diet contained less acetate (*p* < 0.001; Table 3), isobutyrate (*p* ≤ 0.010), and isovalerate (all *p* ≤ 0.022, except Exp 1, *p* = 0.86), more propionate (*p* ≤ 0.003), butyrate (*p* ≤ 0.002), and valerate (*p* ≤ 0.039) and showed a smaller acetate:propionate ratio (*p* ≤ 0.002) than that of cows receiving the M-TNC diet.

In the hay-based Exp 1 and Exp 3, the total volatile fatty acids (VFA) in the ruminal fluid of H-TNC cows tended to be more (*p* = 0.057) and less (*p* = 0.022), respectively, than in M-TNC cows, while total VFA levels were not influenced by diet in the experiment in which fresh cut herbage was fed (Exp 2; *p* = 0.21). In Exp 2 and Exp 3, the ruminal fluid of M-TNC cows contained greater ammonia concentrations (*p* ≤ 0.003) than that of H-TNC cows, while ammonia concentrations were not influenced by diet in Exp 1 (*p* = 0.24). In Exp 3 only, bicarbonate concentrations were greater in the ruminal fluid of H-TNC cows than in M-TNC cows (*p* = 0.013).

#### 3.1.4 Ruminal pH

Across all experiments, the minimum pH of cows receiving the H-TNC diet was lower (*p* ≤ 0.030; Table 4) than that of M-TNC cows. In Exp 2 only, the H-TNC diet additionally reduced and tended to reduce the cows’ mean (*p* = 0.013) and maximum pH (*p* = 0.092), respectively, increased the number of bouts at a pH <5.8 (*p* = 0.012), and tended to increase the time and the duration of bouts with a pH <5.8 compared (*p* ≤ 0.083) to the M-TNC diet. In Exp 1 only, the H-TNC diet tended to increase the time and AUC with a pH <5.5 (*p* ≤ 0.092) in comparison to the M-TNC diet.

**Table 4:** Effects of the experimental diets on ruminal pH by experiment.

	Experiment 1 (n = 8)						Experiment 2 (n = 7)						Experiment 3 (n = 8)				
	Diet		SEM	P value			Diet		SEM	P value			Diet		SEM	P value	
	H-TNC	M-TNC		Diet			H-TNC	M-TNC		Diet			H-TNC	M-TNC		Diet	
Mean pH	5.80	5.87	0.08	0.36			6.12	6.32	0.12	0.032			5.77	5.75	0.07	0.76	
Minimum pH	5.25	5.36	0.05	0.030			5.57	5.80	0.12	0.012			5.19	5.28	0.05	0.026	
Maximum pH	6.65	6.68	0.04	0.54			6.82	6.91	0.07	0.098			6.64	6.55	0.10	0.30	
pH range	1.40	1.33	0.07	0.27			1.25	1.10	0.09	0.11			1.45	1.28	0.12	0.10	
pH < 5.8																	
Time (min/d)	761	693	133	0.58			329	141	132	0.048			731	842	121	0.40	
Bouts (n)	8.88	8.50	1.44	0.94			8.14	4.00	3.34	0.008			9.63	8.75	1.82	0.62	
Duration of bouts (min)	74.9	75.1	25.7	0.72			41.6	22.2	9.05	0.11			95.4	133	35.1	0.32	
AUC (pH × min)	251	181	63.7	0.17			54.1	22.1	24.0	0.12			228	256	45.5	0.55	
pH < 5.5																	
Time (min/d)	424	263	122	0.092			44.6	19.6	24.8	0.35			377	415	81.6	0.65	
Bouts (n)	7.13	5.00	1.17	0.15			2.14	0.57	0.96	0.095			6.50	7.25	1.64	0.73	
Duration of bouts (min)	89.1	33.9	51.3	0.34			17.1	5.53	7.01	0.16			63.9	70.3	12.8	0.62	
AUC (pH × min)	69.6	35.7	26.4	0.069			1.87	1.16	1.40	0.69			59.1	59.6	16.2	0.97	
pH < 5.2																	
Time (min/d)	46.3	21.2	26.7	0.20			0	0	NA	-			37.4	37.7	25.6	0.99	
Bouts (n)	1.13	0.75	0.85	0.67			0	0	NA	-			0.63	1.00	0.55	0.25	
Duration of bouts (min)	49.5	6.01	32.8	0.20			0	0	NA	-			24.4	31.0	22.7	0.78	
AUC (pH × min)	1.61	0.20	0.89	0.11			0	0	NA	-			3.11	1.61	2.36	0.53	

Abbreviations: H-TNC: high sugar, M-TNC: medium sugar, SEM: standard error of the mean, AUC: area under the curve, *Significant effect of period, **Significant effect of sequence. Experiment 1 and 3: hay-based diet with concentrate supplementation; experiment 2: freshly cut herbage-based diet without concentrate supplementation; - not analyzed

#### 3.1.5 Correlations

Across diets, a greater AUC_pH<5.8_ was related to greater intakes of total DM (*r* = 0.41; *p* < 0.05; Table 5), NDF (*r* = 0.62; *p* < 0.05) and TNC (*r* = 0.53; *p* < 0.05), a lower number of DIM (*r* = −0.67; *p* < 0.05), and greater milk yield (*r* = 0.68; *p* < 0.05). For H-TNC cows only, a greater AUC_pH<5.8_ was further associated with greater WSC intake (*r* = 0.55; *p* < 0.05), greater propionate (*r* = 0.42; *p* < 0.05) and valerate (*r* = 0.53; *p* < 0.05) proportions, a lower isobutyrate proportion (*r* = −0.65; *p* < 0.05), and a lower acetate:propionate ratio (*r* = −0.40; *p* < 0.05) in the ruminal fluid.

**Table 5:** Significant (p < 0.05) Pearson correlations with AUCpH< 5.8.

AUC_pH<5.8_ ∼		r (all)	r (H-TNC)	r (M-TNC)
Total DM intake (kg/d)		0.41	0.46	0.48
WSC intake (kg/d)		-	0.55	-
Starch intake (kg/d)		0.63	0.66	0.60
TNC intake (kg/d)		0.53	0.68	0.57
NDF intake (kg/d)		0.62	0.51	0.74
Milk yield (kg/d)		0.68	0.67	0.63
Days in milk		−0.67	−0.67	−0.67
Propionate (mol %)		-	0.42	-
Iso-butyrate (mol %)		-	−0.65	-
Valerate (mol %)		-	0.53	-
Acetate:Propionate		-	−0.40	-

Abbreviations: H-TNC: high sugar, M-TNC: medium sugar, AUC: area under the curve, DM: dry matter, NDF: neutral detergent fibre, TNC: total non-structural carbohydrates, WSC: water soluble carbohydrates.

### 3.2 Between-cow variation: descriptive characterisation of cows with expected and unexpected ruminal pH reactions to increased TNC intake

**Fig. 1:**
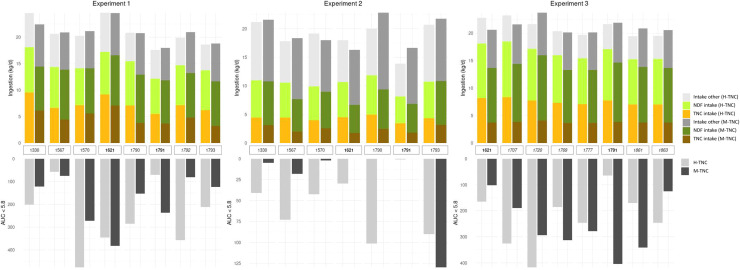
Individual differences of feed intake and AUC_pH<5.8_ (area under the curve at pH <5.8) by cow, experimental diet (moderate-sugar (M-TNC), high-sugar (H-TNC)) and experiment (1,2,3). Numbers of cows that participated in one, two and three experiments are printed in italic, plaine and bold font.

Across the experiments, all cows had a similar DMI, but ingested greater TNC amounts when fed the H-TNC diet compared to the M-TNC diet (Fig. 1). In two-thirds of the observations, that is 14, out of the 23 within-animal crossover observations, cows reacted to the H-TNC diet as commonly expected, namely by increasing the AUC_pH<5.8_ compared to the M-TNC diet (Fig. 1). The AUC_pH<5.8_ of the remaining cows (*n* = 9) reacted to the diets in an unexpected way, i.e. it did either decrease in response to the H-TNC diet or did not vary between experimental diets (Fig. 1).

**Fig. 2:**
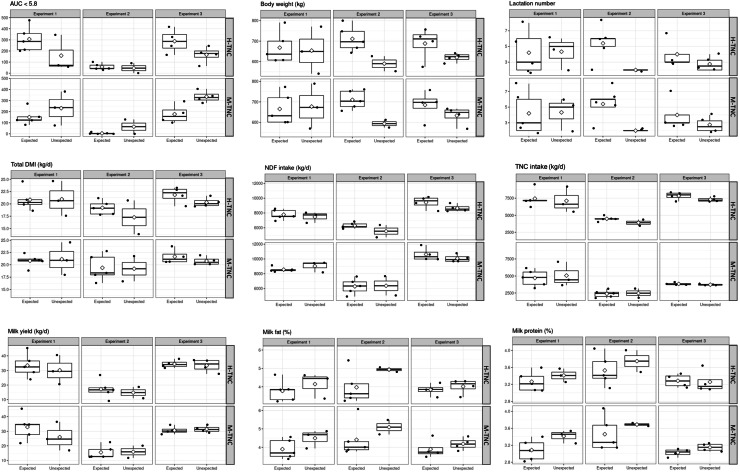
Characteristics of cows with expected and unexpected ruminal pH reactions to the experimental diets. In expectedly reacting cows (n = 14), the AUC_pH<5.8_ (area under the curve at pH <5.8) increased with the H-TNC (high-sugar) diet compared to the M-TNC (moderate-sugar) diet. In unexpectedly reacting cows (n = 9), the AUCpH<5.8 did either decrease under the H-TNC diet or did not vary between experimental diets.

In comparison to cows showing unexpected ruminal pH reactions, the cows with an expected reaction, ingested slightly more total DM (except in Exp 1), NDF, and TNC when receiving the H-TNC diet, but had similar intakes under M-TNC (Fig. 2). Irrespective of the diet, cows with an expected ruminal pH reaction showed a further tendency towards a greater lactation number and had a greater body weight (except for Exp 1) and lower milk fat and protein concentrations than cows with unexpected reactions (Fig. 2).

When comparing individual diet-related changes between the M-TNC and H-TNC diet (ΔH-TNC - M-TNC), cows showing an expected ruminal pH reaction – that is, an increase in AUC_pH<5.8_ in response to the H-TNC diet – had a relatively stable BW, while cows with unexpected reactions had a lower BW when fed the H-TNC than when fed the M-TNC diet (Table 6). In expectedly reacting cows, the greater TNC intake with the H-TNC diet was slightly more pronounced, and the lower NDF intake less pronounced, than in unexpectedly reacting cows. Further, the lower proportions of acetate and isobutyrate and greater proportions of propionate (except for Exp 1) with the H-TNC diet were more pronounced for expectedly reacting than unexpectedly reacting cows. Finally, while expectedly reacting cows had lower bicarbonate levels with the H-TNC diet compared to the M-TNC diet, the inversed trend was observed for unexpectedly reacting animals (Table 6).

**Table 6:** Diet-related changes between the H-TNC and M-TNC diet (ΔH-TNC - M-TNC) of cows with expected and unexpected AUC reactions.

	Experiment 1 (n = 8)				Experiment 2 (n = 7)				Experiment 3 (n = 8)		
	AUC reaction†		SEM		AUC reaction		SEM		AUC reaction		SEM
	Expected	Unexpected			Expected	Unexpected			Expected	Unexpected	
Body weight (kg)	2.89	−23.4	6.15		1.13	−3.75	9.79		3.51	−13.1	7.12
TNC intake (kg/d)	2.74	2.06	0.24		2.10	1.43	2.48		4.01	3.58	1.72
NDF intake (kg/d)	−0.78	−1.55	0.23		0.00	−0.82	3.27		−1.18	−1.42	2.81
Acetate (mol %)	−11.4	−9.04	1.04		−4.07	−3.57	0.34		−6.00	−5.81	1.01
Propionate (mol %)	3.50	3.77	0.88		3.08	1.54	0.52		2.65	1.91	0.70
Iso-butyrate (mol %)	−0.12	−0.07	0.02		−0.27	−0.11	0.10		−0.33	−0.27	0.02
Bicarbonate (mmol/L)	−0.01	0.002	0.002		−0.01	0.001	0.004		0.01	0.02	0.009

Abbreviations: H-TNC: high sugar, M-TNC: medium sugar, SEM: standard error of the mean, AUC: area under the curve, Experiment 1 and 3: hay-based diet with concentrate supplementation; experiment 2: freshly cut herbage-based diet without concentrate supplementation. NDF: neutral detergent fibre, TNC: total non-structural carbohydrates.

† In expectedly reacting cows (n = 14), the AUCpH<5.8 increased with the H-TNC diet compared to the M-TNC diet. In unexpectedly reacting cows (n = 9), the AUCpH<5.8 either decreased under the H-TNC diet or did not vary between experimental diets.

### 3.3 Within-cow variation of ruminal pH reactions across multiple experiments

**Fig. 3:**
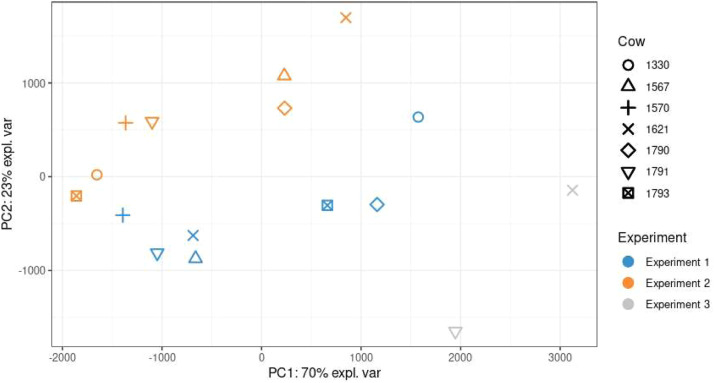
PCA score plot of cows that were present in more than one experiment using individual changes between diets (ΔH-TNC (high-sugar) – M-TNC (moderate-sugar)). Symbols represent individuals as indicated in the legend; experiment 1, 2 and 3 are represented in blue, orange and grey, respectively.

For three out of the seven cows that participated in at least two experiments, the AUC_pH<5.8_ reaction in each Exp was similar; that is, it increased in response to the H-TNC diet. However, the remaining four cows showed alternating reactions to the H-TNC diet (unchanged AUC_pH<5.8_ → increased AUCpH<5.8; decreased AUC_pH<5.8_ → increased AUC_pH<5.8_; decreased AUC_pH<5.8_ → unchanged AUC_pH<5.8_ → decreased AUC_pH<5.8_; increased AUC_pH<5.8_ → decreased AUC_pH<5.8_). When considering the individual changes between diets (ΔH-TNC - M-TNC), a PCA including only cows that participated in at least two experiments showed no clustering by individual cow, but there was a strong separation by experiment (Fig. 3).

## 4 Discussion

### 4.1 General effects of dietary WSC and starch content

In the present study, the greater TNC levels of the H-TNC diet compared to the M-TNC diet were based on greater WSC content in the respective variant of the offered forage, that is, fresh herbage or hay. The effects of greater WSC contents on ruminal pH, the VFA profile, and milk yield, as found in the present study, are in line with earlier works (Gao & Oba, 2016; Miller et al*.*, 2001). However, other studies did not observe increased milk yields, milk protein, ruminal fermentation shifts, or decreased ruminal pH (reviewed by Klevenhusen & Zebeli, 2021). Inconsistent effects of WSC-rich diets have been discussed earlier (Klevenhusen & Zebeli, 2021). It seems that the differing findings relate to different WSC types with different inherent fermentation rates, total fed WSC amounts, and the feed access during the day (ad libitum vs. feed offered once daily), as well as dietary NDF and CP contents in fed diets, which influence the chewing index and ruminal buffering capacity. In addition to these factors, the WSC intake differences between the experimental diets might play a role in the observed effects and their directions. In the present study, the H-TNC cows ingested 1.8–2.6-fold more WSC kg/d than the M-TNC cows, which is much greater compared to previous studies (Klevenhusen & Zebeli, 2021). The size of the effect of the WSC-rich forage on ruminal pH depended further on the type of forage used. A more pronounced decrease of mean ruminal pH due to the H-TNC diet was observed in the experiment with the freshly cut herbage-based diet compared to those with the hay-based diets. This is surprising, since in the herbage-based experiment, the NDF intake was not lower in the H-TNC diet (in contrast to the hay-based experiments), and this should actually have had a positive effect on the ruminal buffer capacity (as mentioned above). It is unclear why the pH-lowering effect of increased TNC intake was more pronounced in the herbage-based diet. We hypothesise that at lower levels of total TNC intake, an increase in TNC has a more pronounced pH-lowering effect than at higher TNC intake levels, where the pH response might be attenuated after having already reached relatively low values (ceiling effect). Another hypothesis is the interaction effects between starch and WSC, which might have attenuated the pH-lowering effect, or a different WSC composition in fresh herbage (higher proportion of disaccharides).

The decrease in ruminal pH by increased starch intake is well known and much more predictable than the WSC-mediated effects. It is caused by the formation of a propionate-dominated fermentation pattern and lactate during starch digestion (Zebeli et al., 2008). This effect was also present in our study. For example, the greater amount of starch, that is, the greater amounts of concentrates distributed to the M-TNC cows compared to H-TNC cows in Exp 3, was probably the main driver in lowering their mean ruminal pH and AUC_pH<5.8_. A greater starch intake may also explain the generally lower and less stable ruminal pH, the longer time_pH<5.8_ (693–842 min/d) and therefore, greater SARA risk, of cows fed hay and concentrates in comparison to cows fed freshly cut herbage without concentrates (time_pH<5.8_: 141–329 min/d). In view of ruminants being originally herbivores, this outcome confirms that a herbage-based diet without concentrate supplementation is a physiological diet, which, at least in terms of ruminal pH, promotes good rumen health.

### 4.2 Inter-individual variations in ruminal pH reactions

Suggestions for SARA thresholds vary from a daily average ruminal pH < 6.16 (Zebeli et al., 2008) and a time of > 3h/d with a ruminal pH <5.6 (Plaizier et al., 2008) to a time of 5–6 h/d with a pH <5.8 (Zebeli et al., 2008). Following these thresholds, the cows in the present study were in SARA conditions in Experiments 1 and 3, that is, the hay-based experiments, independently of the experimental diet and, therefore, the WSC content of hay. As stated in the introduction, SARA can have various consequences for the animal, including reduced DMI, changes in feed intake behaviour, milk production, inflammation, and others. In the present study, there were no indicators of such consequences, similar to previous studies (Zschiesche et al., 2023). The absence of detrimental effects may be related to the relatively short duration of the experiments (6 weeks) and the severity of SARA.

A recommended indicator for the detection and evaluation of SARA is the area under the pH × time curve, as it combines both the duration and amplitude of a pH decrease (Dijkstra et al., 2020). Therefore, we used the AUC_pH<5.8_ in the present work for the detailed analysis of inter- and intra-animal variations. We found that the animals’ reactions in terms of AUC_pH<5.8_ to increased dietary TNC contents increased in two-thirds of the observations, whereas in one third, the AUC_pH<5.8_ was not affected or decreased. This finding confirms the inter-individual variability of ruminal pH reactions reported earlier, with 40 % (31/78 cows; Nasrollahi et al. (2017)) and 70 % (11/16 cows; Humer et al. (2015)) of cows showing a tendency to lower their pH in response to high-concentrate diets. In the present study, we labelled animals whose AUC_pH<5.8_ increased with the H-TNC diet as reacting in an expected way. By contrast, the animals whose AUC_pH<5.8_ was decreased or unaffected by the H-TNC diet were labelled as reacting in an unexpected way. This designation (expected vs. unexpected) was based on the overall observed tendency of the H-TNC diet to decrease the ruminal pH minimum. In Exp 2 and Exp 3, animals reacting in an expected way were characterised – under the H-TNC diet – by numerical greater DM, NDF, and TNC intakes, numerical greater body weight and lactation number.

The positive association between SARA susceptibility and feed intake is in line with an earlier observatory on-farm study on early-lactating cows (Zschiesche et al., 2023) and a study provoking SARA in mid-lactating cows (Neubauer et al., 2020). In an experiment with 13 cows and a large amount (153) of observations per cow, feed intake explained most of the 38 % variance in ruminal pH (Mensching et al., 2020). Other studies did not find this link (Humer et al., 2015; Jing et al., 2018; Khiaosa-ard et al., 2018; Nasrollahi et al., 2017). In view of the lack of association in Experiment 1 and the unanimous literature, we might posit that feed intake and body size are not pertinent factors in drawing any general conclusion about SARA susceptibility or SARA risk. Other explanatory factors for SARA susceptibility or tolerance have been described, including the behaviour of feed intake and rumination, such as feed sorting, intake rate and meal size (Humer et al., 2015; Mensching et al., 2020), which were not assessed in the present study. However, the explained variance of such factors in ruminal pH seems limited. For example, the time spent on feed intake and rumination and the rumination frequency explained <5 % of the variance in ruminal pH in an on-farm study with 100 cows (Zschiesche et al., 2023). Therefore, both feeding and rumination behaviour and feed intake seem relevant for ruminal pH variations, but only to a limited degree.

Another factor that influences ruminal pH reactions might relate to the physiology of the animal itself. The lower increase in ruminal bicarbonate concentrations of expectedly reacting animals compared to unexpectedly reacting animals suggest that nutritional physiological processes may influence SARA susceptibility. Consequently, SARA susceptibility may be related to a lower ruminal buffer capacity or rates. Interestingly, the cows that reacted in an unexpected manner, irrespective of the experimental diet, had consistently greater percentages of milk fat and, except for H-TNC in Exp 3, protein than those that reacted as expected. This finding is in line with so-called SARA-tolerant cows, which tend to have greater milk fat and protein contents (Khiaosa-ard et al., 2018; Nasrollahi et al., 2017); the underlying reasons might be genetic or related to rumen health. In the present study, no analyses of ruminal microbiota or other omics approaches were conducted; future studies should consider such analyses to gain deeper insight into the physiological factors contributing to SARA susceptibility.

Most studies investigating the susceptibility of cows to SARA categorised them as either SARA susceptible or tolerant based on their pH response under a certain diet (in most cases, high-concentrate) (Humer et al., 2015; Jing et al., 2018; Khiaosa-ard et al., 2018). The present study was a crossover study, indicating that data were collected from animals under both M-TNC and H-TNC conditions. Retrospectively, we categorised the animals based on the difference in AUC_pH<5.8_ between the two experimental diet (ΔH-TNC - M-TNC), that is, by their absolute ruminal pH × time reaction to increased dietary amounts of sugar. To our knowledge, only one other study used a similar approach. In that study, 12 cows were fed weekly alternating control and SARA-provoking diets over 5 weeks (Khiaosa-ard et al., 2018). Here, the SARA susceptible animals (*n* = 6, the six cows with the greatest pH suppression across the 5 experimental weeks) had lower pH levels than the tolerant (*n* = 6, the six remaining cows) throughout the experiment, independently of the diet fed. It is interesting that in the present study, the unexpectedly reacting—in other studies, they might have been named “tolerant”—animals had lower ruminal pH, AUC_pH<5.8_, and time_pH<5.8_ when fed the H-TNC diet but greater levels of the latter variables when fed the M-TNC diet, compared to the expectedly reacting—or “susceptible”—cows. This finding questions the categorisation of animals as either SARA-susceptible or SARA-tolerant. The ruminal pH of the animals that reacted as expected was more sensitive to the H-TNC diet, that is, when greater amounts of TNC were ingested. Cows that had unexpected reactions seemed less sensitive to greater TNC amounts but coped less well in terms of ruminal pH with the M-TNC diet.

As mentioned earlier in the section discussing the pronounced pH-lowering effect at low TNC intake levels, we again hypothesise that the level of TNC intake plays a role in the extent of the pH reaction. Furthermore, individuals may differ in their sensitivity to varying TNC intake levels. Finally, the varying sensitivity might also relate to the sequence in which the animals received the experimental diets. In total, 64 % of the animals that reacted as expected (i.e. 9 out of 14) received the M-TNC diet first before the H-TNC diet, whereas 77 % of those with unexpected reactions (7 out of 9) received the H-TNC diet first before the M-TNC diet. This tendency was mostly present in the hay-based experiments (Exp 1 and Exp 3), and to a lesser extent in Exp 2. This might suggest that receiving a TNC-rich diet over 6 weeks sensitised (or damaged) the rumen environment to such a point that a subsequent diet with lower TNC amounts did not alleviate a decreased ruminal pH.

In view of the discussed points, it is also unclear whether some of the animals were “better adapted” or even more tolerant/robust in terms of rumen health under varying dietary sugar levels and, if so, when the adaptation occurred.

### 4.3 Intra-individual variations in ruminal pH reactions

This study uniquely tracked individual cows across multiple experiments, allowing to investigate intra-individual variation. More than half of the animals that underwent several experiments did not show consistent ruminal pH reactions to H-TNC diets. This is not surprising, as the ruminal pH and its range can vary considerably for individual cows, for example, when ruminal fluid is repeatedly sampled over 54 h (Palmonari et al., 2010). A previous study showed that the pH reaction to a SARA challenge was aggravated when the animal had already experienced a SARA episode in the past (Dohme et al., 2008). Intra-individual variation might thus be diet-dependent, time-dependent, and influenced by previous experience. These variations are hard to consider in practice and research protocols, as it is difficult to identify a priori the animals that are at risk for SARA when fed a certain diet at a certain time point in their lives. Thus, to follow up on SARA risk in individual animals, it is necessary to conduct individual measurements focusing on pH variation and deviations from individual levels rather than thresholds (Heirbaut et al., 2022).

## 5 Conclusion

Diets rich in TNC may cause ruminal pH depression in some animals but not in others, with responses varying both between and within individuals over time. These inter- and intra-animal variations appear to be influenced by animal characteristics such as ingestion capacity, dietary TNC levels, and previous dietary experience. Further research, including for example, microbial and other omics analyses, is needed to better understand the complex relationships and interactions underlying these variations. As a result, it remains challenging to identify animals at risk of subacute ruminal acidosis (SARA) in advance. Reliable assessment of SARA therefore requires continuous monitoring of ruminal pH at the individual level using relative thresholds, as no consistent proxy markers are currently available. Given the limited number of animals in this study, the findings should be interpreted with caution and confirmed through larger, more comprehensive experiments.

## Data and model availability statement

**None of the data were deposited in an official repository, but they are available upon request.**

## Financial support statement

**This research received no specific grant from any funding agency, commercial or not-for-profit section.**

## Declaration of generative AI and AI-assisted technologies in the writing process

During the preparation of this work the authors used ChatGPT in order to improve the language of the manuscript. After using this tool, the authors reviewed and edited the content as needed and take full responsibility for the content of the published article.

## Ethical statement

The experiments were in accordance with the Swiss laws of animal protection and were approved by the cantonal veterinary office of Fribourg, Switzerland (national No 18,630, 19,471, and 21,789).

## CRediT authorship contribution statement

**Anna-Maria Reiche:** Writing – original draft, Visualization, Formal analysis. **Andreas Münger:** Writing – review & editing, Visualization, Methodology, Investigation, Data curation, Conceptualization. **Frigga Dohme-Meier:** Writing – review & editing, Resources, Project administration, Conceptualization.

## Declaration of competing interest

The authors declare that they have no known competing financial interests or personal relationships that could have appeared to influence the work reported in this paper.
